# Pancytopenia in HIV-positive patients with congestive heart failure: mechanisms, diagnosis, and treatment approaches

**DOI:** 10.1097/MS9.0000000000003501

**Published:** 2025-06-16

**Authors:** Emmanuel Ifeanyi Obeagu, Olga G. Goryacheva

**Affiliations:** a Department of Biomedical and Laboratory Science, Africa University, Mutare, Zimbabwe; b Department of Polyclinic Therapy, Perm State Medical University named after Academician E.A. Wagner, Ministry of Health of the Russian Federation, Perm, Russian Federation

**Keywords:** antiretroviral therapy, bone marrow suppression, congestive heart failure, HIV, pancytopenia

## Abstract

Pancytopenia, characterized by the simultaneous reduction of red blood cells, white blood cells, and platelets, is a significant hematological complication in individuals living with human immunodeficiency virus (HIV). Its presence often reflects disease progression, immune suppression, and concurrent opportunistic infections. When pancytopenia coexists with congestive heart failure (CHF) – a condition increasingly prevalent among HIV-positive patients due to chronic inflammation, antiretroviral therapy (ART)-associated cardiotoxicity, and aging – the clinical burden intensifies, leading to diagnostic and therapeutic dilemmas. This review aims to examine the underlying mechanisms, diagnostic challenges, and management strategies of pancytopenia in HIV-positive patients with CHF. It explores the multifactorial pathophysiology, including direct viral effects on the bone marrow, drug-induced cytopenias, opportunistic infections, nutritional deficiencies, and CHF-related impairments in erythropoietin and thrombopoietin production. Additionally, the review highlights overlapping symptoms that complicate diagnosis and evaluates current treatment options ranging from ART optimization to hematopoietic growth factors and supportive care.

## Introduction

Pancytopenia, defined as the concurrent reduction in red blood cells, white blood cells, and platelets, is a significant hematological abnormality observed in various clinical settings[1]. In individuals with human immunodeficiency virus (HIV) infection, the prevalence of pancytopenia has been reported to be approximately 8.7%, with affected patients exhibiting lower body mass index, reduced CD4 counts, and higher HIV viral loads compared to those without pancytopenia. The etiology of pancytopenia in HIV-positive patients is multifactorial, encompassing direct viral effects on bone marrow, opportunistic infections, malignancies, and adverse reactions to antiretroviral therapy (ART).^[2-5]^ Congestive heart failure (CHF) is increasingly recognized as a comorbidity in the HIV-positive population[6]. Studies have indicated that individuals with HIV are at an elevated risk of developing heart failure, with mechanisms including chronic inflammation, immune activation, and direct myocardial effects of the virus contributing to this increased susceptibility.^[6-9]^ The coexistence of HIV and CHF presents a complex clinical scenario, as both conditions independently contribute to hematological abnormalities, and their interplay may exacerbate the severity of pancytopenia[10]. The pathophysiology of pancytopenia in HIV-positive patients with CHF involves a combination of factors[11]. Direct HIV infection of bone marrow progenitor cells can lead to impaired hematopoiesis, while chronic immune activation results in elevated cytokine levels, further disrupting bone marrow function. Additionally, ART, particularly regimens including zidovudine, has been associated with bone marrow suppression. In the context of CHF, reduced cardiac output and subsequent tissue hypoxia may impair bone marrow perfusion, compounding hematopoietic dysfunction. Moreover, the use of certain cardiovascular medications can contribute to cytopenias, adding another layer of complexity to patient management.^[12-16]^

**HIGHLIGHTS**

- Pancytopenia in HIV results from bone marrow suppression, opportunistic infections, and antiretroviral toxicity.
- Congestive heart failure (CHF) worsens hematologic abnormalities through chronic inflammation and reduced oxygen delivery.
- Diagnosis requires complete blood count, bone marrow biopsy, and infectious screening.
- Treatment involves antiretroviral adjustments, transfusions, and heart failure management.
- Future research should explore targeted therapies addressing both hematologic and cardiac complications.

Diagnosing pancytopenia in this patient population necessitates a comprehensive approach. Initial laboratory evaluations should include a complete blood count (CBC) with differential, reticulocyte count, and peripheral blood smear to assess the degree and lineage of cytopenias[17]. Bone marrow aspiration and biopsy are often warranted to evaluate marrow cellularity and identify potential infiltrative processes. Infectious workup is essential, as pancytopenia can be associated with infections such as HIV, malaria, and tuberculosis[18]. Additionally, assessment of vitamin B12 and folate levels, liver function tests, and lactate dehydrogenase can aid in identifying reversible causes of pancytopenia. Management strategies should be tailored to the underlying etiology of pancytopenia. Optimization of ART is crucial, with consideration given to modifying regimens to minimize myelosuppressive effects.^[19-21]^ Addressing nutritional deficiencies through supplementation of iron, vitamin B12, and folate is essential. Treatment of opportunistic infections, such as initiating therapy for tuberculosis or cytomegalovirus, should be promptly undertaken. In cases where autoimmune conditions or malignancies are identified, appropriate therapeutic interventions should be implemented. Supportive care measures, including transfusions and growth factor support, may be necessary in severe cases to manage symptomatic cytopenias[22].

## Aim

This review aims to explore the mechanisms, diagnostic approaches, and treatment strategies for pancytopenia in HIV-positive patients with CHF.

## Identified gaps in the literature

- Limited studies on the co-occurrence of pancytopenia, HIV, and CHF:Most existing literature addresses pancytopenia in HIV or CHF separately. There is a lack of focused clinical or epidemiological studies examining patients who suffer from all three conditions concurrently. This hinders the ability to understand prevalence, prognosis, and optimal interventions for this unique patient subgroup.
- Insufficient mechanistic insights into combined pathophysiology:While bone marrow suppression in HIV and impaired erythropoiesis in CHF are individually documented, little is known about how these mechanisms may interact or compound one another. Synergistic effects of immune activation, marrow suppression, and circulatory dysfunction in this patient population remain poorly characterized.
- Scarcity of tailored diagnostic algorithms and management guidelines:Current clinical guidelines treat pancytopenia, HIV, and CHF independently. There are no integrated diagnostic or therapeutic pathways that account for overlapping pharmacologic toxicity, nutritional deficiencies, infection risk, and cardiac complications in HIV-positive patients with CHF and pancytopenia.
- Lack of prospective and interventional studies:Most data come from retrospective analyses or case reports. There is a need for prospective cohort studies and randomized controlled trials (RCTs) to evaluate the safety and efficacy of treatment options like hematopoietic growth factors or modified ART regimens in this subgroup.
- Underrepresentation in clinical trials:HIV-positive individuals with heart failure and pancytopenia are often excluded from trials due to comorbidities. This contributes to a paucity of evidence on the safety of common CHF treatments and hematologic interventions in this population.

## Review methods

This narrative review was conducted to explore and synthesize existing knowledge on the pathophysiological mechanisms, diagnostic considerations, and therapeutic strategies for pancytopenia in HIV-positive patients with CHF. The objective was to provide a comprehensive understanding of this complex clinical condition and to identify areas requiring further research or clinical innovation. To achieve this, a structured yet flexible literature search strategy was employed. Relevant articles were retrieved from multiple electronic databases, including PubMed, Google Scholar, ScienceDirect, and Scopus. The search focused on studies published from January 2000 to March 2025, to ensure inclusion of both foundational knowledge and the most recent advances in the field. This 25-year window was chosen to capture the evolution of antiretroviral therapy (ART), changing trends in CHF management, and developments in hematologic understanding relevant to pancytopenia.

A combination of Medical Subject Headings terms and free-text keywords was used to maximize retrieval of relevant articles. The primary keywords and their Boolean combinations included:

- “Pancytopenia” AND “HIV”
- “Pancytopenia” AND “Congestive Heart Failure”
- “Hematologic abnormalities” AND “HIV-positive patients”
- “Cytopenias in HIV”
- “HIV-associated cytopenias” AND “CHF”
- “Heart failure” AND “immune suppression”
- “Antiretroviral therapy” AND “bone marrow suppression”
- “HIV” AND “thrombocytopenia” OR “anemia” OR “neutropenia”
- “Individualized treatment” AND “HIV” AND “cardiovascular disease”
- “Long-term management” AND “HIV and CHF”

Filters were applied to select peer-reviewed original research articles, clinical trials, meta-analyses, and high-quality review articles written in English. Studies involving both adult and pediatric populations were initially considered; however, the review primarily focused on adult populations to maintain specificity. Articles were screened for relevance based on titles and abstracts, followed by a thorough full-text review of selected publications. Additional studies were identified by manually searching the reference lists of key articles. Priority was given to studies that provided insights into the mechanistic basis of pancytopenia, the clinical impact of coexisting HIV and CHF, and evidence-based diagnostic and treatment protocols. Where appropriate, national and international guidelines – such as those from the World Health Organization, Centers for Disease Control and Prevention, and American Heart Association – were also reviewed to align the discussion with globally accepted standards of care.

### Limitations of the review

While this narrative review aimed to provide a comprehensive overview of pancytopenia in HIV-positive patients with CHF, several limitations must be acknowledged. First, the search was limited to articles published in English and focused on studies from 2000 to 2025, which may have excluded relevant research in other languages or older studies that could contribute valuable insights. Furthermore, the reliance on electronic databases – primarily PubMed, Google Scholar, ScienceDirect, and Scopus – may have missed studies published in less widely indexed journals or in grey literature, such as conference abstracts or government reports. Additionally, despite efforts to include a wide range of studies, the review primarily focuses on adult populations, limiting its applicability to pediatric or adolescent patients. The complex nature of pancytopenia in HIV/CHF patients, compounded by varying treatment regimens, comorbidities, and socio-economic factors, makes it difficult to generalize findings universally. Consequently, the findings may not fully represent all regional or demographic variations in disease presentation and management. Finally, the heterogeneity of the studies included in the review, with variations in study design, sample size, and methodology, poses a challenge for drawing definitive conclusions. While this review highlights broad trends and evidence-based approaches, it may not capture the full complexity of individual patient experiences or the most nuanced aspects of clinical practice.

## Mechanisms of pancytopenia in HIV-CHF patients

Pancytopenia, the simultaneous reduction of red blood cells, white blood cells, and platelets, presents a significant challenge in HIV-positive patients with coexisting CHF. The convergence of these two chronic conditions creates a complex pathophysiological environment that disrupts normal hematopoiesis and leads to bone marrow dysfunction. This interplay is driven by several interconnected mechanisms, including direct viral effects on the bone marrow, CHF-induced hypoxia and inflammation, opportunistic infections, medication-related toxicity, nutritional deficiencies, and increased peripheral destruction of blood cells[23].

### Direct effects of HIV on the bone marrow

One of the primary contributors to pancytopenia in HIV-CHF patients is the direct impact of the HIV virus on the bone marrow. HIV can infect CD34+ hematopoietic progenitor cells, impairing their ability to differentiate into mature blood cells. Additionally, HIV disrupts the bone marrow microenvironment by infecting stromal cells, which play a critical role in supporting hematopoiesis. This results in a dysfunctional hematopoietic niche, leading to inadequate blood cell production. Furthermore, HIV induces chronic immune activation, triggering the release of pro-inflammatory cytokines such as tumor necrosis factor-alpha (TNF-α), interleukin-1 (IL-1), and interferon-gamma (IFN-γ). These inflammatory mediators exert suppressive effects on the bone marrow, reducing its ability to generate red blood cells (causing anemia), white blood cells (leading to leukopenia), and platelets (resulting in thrombocytopenia). Over time, this chronic inflammation can lead to bone marrow fibrosis, further limiting hematopoietic capacity[24].

### Congestive heart failure and bone marrow dysfunction

In addition to the direct effects of HIV, CHF further exacerbates pancytopenia by impairing oxygen delivery to the bone marrow. As CHF progresses, reduced cardiac output and systemic congestion lead to tissue hypoxia. The bone marrow, being highly oxygen-dependent, becomes increasingly dysfunctional under these conditions, leading to ineffective hematopoiesis. CHF also contributes to systemic inflammation, characterized by elevated levels of inflammatory markers such as C-reactive protein (CRP), interleukin-6 (IL-6), and TNF-α. These pro-inflammatory cytokines not only inhibit bone marrow function but also promote erythrophagocytosis, where red blood cells are prematurely destroyed by macrophages, further worsening anemia. Another major complication of CHF is renal dysfunction, which plays a key role in worsening anemia. The kidneys produce erythropoietin (EPO), a crucial hormone that stimulates red blood cell production. However, in CHF, renal hypoperfusion and damage lead to decreased erythropoietin levels, reducing erythropoiesis and exacerbating anemia. Additionally, bone marrow congestion due to venous stasis can create a hypoxic and inflammatory environment that further impairs hematopoiesis[25].

### Opportunistic infections and immune-mediated bone marrow suppression

HIV patients, particularly those with CHF, are at increased risk of opportunistic infections, many of which can directly affect the bone marrow. Mycobacterium tuberculosis is a common cause of granulomatous inflammation in the bone marrow, leading to bone marrow necrosis and suppression of blood cell production. Similarly, cytomegalovirus (CMV) and Epstein-Barr virus (EBV) can cause hemophagocytic syndrome, a severe condition characterized by uncontrolled immune activation and destruction of blood cells. Parvovirus B19, another opportunistic infection, is particularly significant as it targets erythroid progenitor cells, leading to pure red cell aplasia and profound anemia. Fungal infections such as histoplasmosis and cryptococcosis can also infiltrate the bone marrow, further compromising its function[26].

### Medication-induced pancytopenia

Both ART and cardiovascular medications contribute to hematological abnormalities in HIV-CHF patients. Among antiretroviral drugs, zidovudine (AZT) is one of the most notorious for causing bone marrow suppression, leading to macrocytic anemia, neutropenia, and thrombocytopenia. Other nucleoside reverse transcriptase inhibitors (NRTIs), such as stavudine (d4T) and lamivudine (3TC), contribute to mitochondrial toxicity, further impairing hematopoiesis. Protease inhibitors, such as ritonavir and lopinavir, have been linked to immune-mediated thrombocytopenia, where platelets are prematurely destroyed. Additionally, drug interactions between ART and CHF medications can worsen pancytopenia. Several cardiovascular drugs used to manage CHF can also contribute to cytopenias. Angiotensin-converting enzyme (ACE) inhibitors, such as lisinopril and enalapril, can suppress erythropoiesis by reducing erythropoietin levels. Beta-blockers, including carvedilol and metoprolol, have been associated with neutropenia and thrombocytopenia, while diuretics, such as furosemide and spironolactone, can cause electrolyte imbalances that indirectly affect hematopoiesis[27].

### Nutritional deficiencies and metabolic abnormalities

HIV and CHF are both associated with malabsorption and metabolic abnormalities, which can contribute to pancytopenia. Patients often develop deficiencies in vitamin B12, folate, and iron, all of which are essential for normal blood cell production. CHF-related gut edema and HIV-induced enteropathy further exacerbate malabsorption, making it difficult for the body to maintain adequate nutrient levels. Iron metabolism is also dysregulated in these patients due to elevated hepcidin levels, a consequence of chronic inflammation. Hepcidin inhibits iron absorption and sequestration, leading to functional iron deficiency anemia despite adequate iron stores. In advanced CHF, patients may develop chronic kidney disease (CKD), which contributes to uremia-induced bone marrow suppression, leading to normocytic, normochromic anemia[28].

### Increased peripheral destruction of blood cells

Beyond bone marrow suppression, HIV-CHF patients may experience accelerated peripheral destruction of blood cells due to immune-mediated mechanisms. Autoimmune hemolytic anemia (AIHA) can develop due to HIV-induced immune dysregulation, leading to increased destruction of red blood cells. Similarly, immune thrombocytopenic purpura (ITP) can cause excessive platelet destruction, resulting in thrombocytopenia and increased bleeding risk. In some cases, CHF-related hepatic congestion and portal hypertension can lead to hypersplenism, a condition where an enlarged spleen excessively sequesters and destroys red blood cells, white blood cells, and platelets. This contributes to the worsening of pancytopenia and increases the risk of infections and bleeding complications[29].

## Diagnosis of pancytopenia in HIV-CHF patients

The diagnosis of pancytopenia in HIV-positive patients with CHF is a complex and multifaceted process. Given the interplay of viral effects, chronic inflammation, medication toxicity, and nutritional deficiencies, identifying the underlying causes of pancytopenia requires a systematic and comprehensive approach. This process involves a thorough clinical evaluation, detailed laboratory investigations, and specialized diagnostic procedures aimed at distinguishing the primary contributors to cytopenia[17].

### Clinical evaluation: identifying key symptoms and risk factors

The initial step in diagnosing pancytopenia in HIV-CHF patients begins with a detailed history and physical examination. Patients often present with fatigue, pallor, shortness of breath, recurrent infections, and easy bruising or bleeding, reflecting the reductions in red blood cells (anemia), white blood cells (leukopenia), and platelets (thrombocytopenia). A history of HIV duration, ART use, opportunistic infections, and CHF severity provides essential clues to the etiology of pancytopenia. The presence of weight loss, night sweats, and chronic diarrhea may suggest underlying opportunistic infections or malignancies affecting the bone marrow, while orthopnea, leg swelling, and exertional dyspnea point toward CHF-related hypoxia and organ congestion. Additionally, a history of medications is crucial. AZT and other nucleoside reverse transcriptase inhibitors (NRTIs) are known for their bone marrow suppressive effects, while CHF medications such as ACE inhibitors and beta-blockers can also contribute to cytopenias. Patients with a history of recurrent blood transfusions may be at risk for iron overload or alloimmunization, both of which can impact hematopoiesis. A thorough physical examination often reveals pallor (from anemia), petechiae or ecchymosis (from thrombocytopenia), and lymphadenopathy or hepatosplenomegaly, which may indicate opportunistic infections, hematologic malignancies, or hypersplenism due to CHF-related hepatic congestion. The presence of cardiac murmurs, jugular venous distension, and lower limb edema further supports CHF as a contributing factor^[17,30]^.

### Complete blood count and peripheral blood smear

The cornerstone of pancytopenia diagnosis is a CBC with differential, which confirms reductions in all three cell lines – red blood cells, white blood cells, and platelets. Anemia is often normocytic or macrocytic, particularly in patients on zidovudine or those with vitamin B12 and folate deficiencies. The reticulocyte count helps differentiate between bone marrow failure (low reticulocyte count) and peripheral destruction (high reticulocyte count, as seen in hemolysis). A peripheral blood smear provides valuable diagnostic insights. Findings such as macrocytosis (in zidovudine toxicity), schistocytes (suggestive of microangiopathic hemolytic anemia), hypersegmented neutrophils (in vitamin B12 or folate deficiency), and giant platelets (in immune thrombocytopenia) guide further workup. The presence of blast cells may indicate leukemia or myelodysplastic syndrome, while intracellular parasites such as Histoplasma or Leishmania suggest bone marrow infections[31].

### Bone marrow examination

If initial investigations are inconclusive, a bone marrow aspiration and biopsy is often necessary to determine the cause of pancytopenia. Bone marrow hypocellularity suggests HIV-related marrow suppression, zidovudine toxicity, or aplastic anemia, whereas hypercellular marrow with dysplastic features raises suspicion for myelodysplastic syndromes (MDS) or opportunistic infections like tuberculosis or fungal infiltration. Bone marrow cultures and special stains (e.g., Ziehl-Neelsen stain for tuberculosis, Giemsa stain for Leishmania, and fungal stains for Cryptococcus and Histoplasma) help identify opportunistic pathogens. Hemophagocytosis, characterized by macrophages engulfing blood cells, suggests hemophagocytic lymphohistiocytosis (HLH), a severe inflammatory condition associated with HIV and CHF[30].

### Serum biochemical tests

Given the high prevalence of nutritional deficiencies in HIV-CHF patients, serum levels of vitamin B12, folate, and iron studies (serum ferritin, transferrin saturation, total iron-binding capacity) are crucial. Low B12 or folate points to nutritional anemia, whereas iron overload (from multiple transfusions) or functional iron deficiency (due to elevated hepcidin in chronic inflammation) can contribute to anemia. Renal and liver function tests assess organ dysfunction, which is common in both HIV and CHF. Elevated creatinine and reduced glomerular filtration rate (GFR) suggest chronic kidney disease-related anemia, while abnormal liver enzymes in a patient with hepatomegaly and ascites may indicate hepatic congestion due to CHF, contributing to hypersplenism and pancytopenia[32].

### Inflammatory and autoimmune markers

Since chronic inflammation plays a significant role in HIV and CHF-associated cytopenias, measuring inflammatory markers such as C-reactive protein (CRP), erythrocyte sedimentation rate (ESR), interleukin-6 (IL-6), and tumor necrosis factor-alpha (TNF-α) can help assess systemic inflammation. Autoimmune mechanisms also contribute to cytopenias in HIV, necessitating tests for autoimmune hemolytic anemia (direct Coombs test), immune thrombocytopenia (platelet autoantibodies), and lupus-related cytopenias (antinuclear antibodies, anti-dsDNA, antiphospholipid antibodies)[31].

#### Viral and infectious workup

Since opportunistic infections can infiltrate the bone marrow and contribute to pancytopenia, targeted microbiological tests are essential. These include:

- HIV viral load and CD4 count to assess immune suppression.
- Blood cultures for Mycobacterium tuberculosis, Cryptococcus, and bacterial infections.
- Polymerase chain reaction (PCR) for parvovirus B19, CMV, and Epstein-Barr virus (EBV).
- Fungal antigen tests (e.g., Histoplasma and Aspergillus galactomannan assay).

In patients with persistent fevers, weight loss, or lymphadenopathy, a bone marrow biopsy with acid-fast bacilli (AFB) staining and fungal cultures is recommended to rule out disseminated tuberculosis or fungal infections[33].

#### Cardiac and hemodynamic assessment

Since CHF contributes to hypoxia, organ congestion, and bone marrow dysfunction, a cardiac workup is crucial.

- Echocardiography assesses ejection fraction (EF), ventricular dysfunction, and pulmonary hypertension, which can contribute to systemic hypoxia and worsen pancytopenia.
- B-type natriuretic peptide (BNP) or N-terminal pro-BNP (NT-proBNP) helps evaluate CHF severity.
- Arterial blood gas (ABG) analysis determines if chronic hypoxia is impairing bone marrow function[34].

## Treatment approaches for pancytopenia in HIV-CHF patients

- Granulocyte colony-stimulating factor (G-CSF, filgrastim) can be used in patients with severe neutropenia (absolute neutrophil count <500 cells/µL) who are at high risk of infections.
- Prophylactic antibiotics (e.g., trimethoprim-sulfamethoxazole for Pneumocystis jirovecii pneumonia) are recommended for patients with profound immunosuppression (CD4 < 200 cells/µL).
- Strict infection control measures (hand hygiene, avoiding crowded places, and timely vaccinations) are crucial in preventing life-threatening bacterial and fungal infections[35].

### Managing thrombocytopenia: reducing bleeding risk and immune modulation

Thrombocytopenia in HIV-positive patients with CHF presents a dual threat: increased risk of bleeding and impaired immune response. Management must be both preventive and corrective, targeting underlying causes while mitigating immediate complications. Treatment options are selected based on severity, etiology, and patient comorbidities, with a strong emphasis on preserving hemostatic balance and immune integrity. ART Optimization is foundational, as effective viral suppression reduces immune-mediated platelet destruction and bone marrow suppression. For instance, AZT is known to exacerbate cytopenias; hence, switching to less myelotoxic regimens (e.g., tenofovir-based therapies) may restore platelet counts. The rationale is that controlling HIV replication minimizes immune dysregulation and autoimmune thrombocytopenia, improving marrow function and reducing platelet consumption. Corticosteroids and intravenous immunoglobulin (IVIG) are indicated in cases of immune thrombocytopenic purpura (ITP) linked to HIV, where autoantibodies target platelets. These agents act by modulating immune responses: corticosteroids reduce autoantibody production, while IVIG saturates Fc receptors on macrophages, preventing platelet destruction. Their use is justified when bleeding risk is high or platelet counts fall below critical thresholds[35]. Thrombopoietin receptor agonists (e.g., eltrombopag, romiplostim) stimulate megakaryocyte proliferation and platelet production, especially in patients with inadequate marrow response. These agents are useful in refractory cases where ART and immunosuppression fail. Their significance lies in directly addressing insufficient platelet generation, particularly in CHF patients with impaired marrow perfusion. Platelet transfusions are reserved for severe thrombocytopenia (<10 000/μL) or active bleeding. Though they provide immediate hemostatic correction, their short-lived effect and risk of alloimmunization limit routine use. In CHF, volume overload must also be considered, making transfusions a careful balancing act. Treatment of secondary causes – such as opportunistic infections (e.g., CMV, tuberculosis), nutritional deficiencies (B12, folate), or drug-induced thrombocytopenia – is critical. Addressing these factors removes persistent triggers for platelet destruction or production failure, providing a long-term solution[35].

### Long-term management and monitoring

#### Long-term management and monitoring

Long-term management and monitoring of pancytopenia in HIV-positive patients with CHF require a dynamic, integrative approach that addresses both clinical manifestations and laboratory abnormalities. This population is at heightened risk for chronic complications due to the cumulative effects of immunosuppression, antiretroviral and cardiovascular drug toxicities, bone marrow dysfunction, and systemic inflammation. As such, continuous surveillance and individualized treatment strategies are essential to improve prognosis and quality of life. Clinically, patients should be regularly assessed for symptoms related to all three cytopenias – fatigue, pallor, and dyspnea (suggestive of anemia); fever, recurrent infections, and oral ulcers (linked to neutropenia); and petechiae, ecchymosis, or mucosal bleeding (indicative of thrombocytopenia). The coexistence of CHF complicates these manifestations: dyspnea, for instance, could stem from either anemia or worsening heart failure, necessitating a nuanced clinical evaluation. Moreover, any signs of fluid overload, reduced exercise tolerance, or arrhythmias should be promptly investigated, as they may point to deteriorating cardiac function aggravated by reduced oxygen-carrying capacity from anemia[17]. Close attention should also be paid to drug-related adverse effects. Several antiretroviral agents, particularly zidovudine, and cardiovascular medications like ACE inhibitors or beta-blockers may contribute to marrow suppression or electrolyte imbalances. Hence, medication regimens must be periodically reviewed and adjusted to minimize iatrogenic cytopenias without compromising virologic or cardiac control. Multidisciplinary collaboration among infectious disease specialists, hematologists, and cardiologists is vital in this regard[30].

*Laboratory monitoring* is equally critical. CBC with differential should be performed routinely – initially biweekly or monthly, then quarterly as the patient stabilizes. Reticulocyte counts help assess bone marrow response in anemic patients, while peripheral blood smears can reveal morphological clues such as megaloblastic changes, blasts, or platelet clumping. Serial viral load and CD4+ T-cell counts are indispensable in evaluating immune recovery and virologic suppression, both of which correlate with improved marrow function over time[31]. Additional investigations include serum vitamin B12, folate, and iron studies to rule out concurrent nutritional deficiencies, which are common in HIV-positive individuals. Bone marrow aspiration and biopsy may be warranted in cases of unexplained persistent pancytopenia, particularly to rule out infiltrative diseases, malignancies (like lymphoma or leukemia), or myelodysplastic syndromes. In the context of CHF, regular monitoring of renal function, NT-proBNP, and echocardiography helps guide heart failure management and fluid balance, which indirectly impacts hematopoietic homeostasis^[32,33]^. Furthermore, markers of inflammation (e.g., CRP, IL-6) and immune activation may be useful in research or advanced clinical settings to better understand chronic marrow suppression in this population. For those receiving growth factors such as erythropoietin or granulocyte colony-stimulating factor (G-CSF), response should be tracked with both laboratory parameters and clinical indicators of improved oxygenation and infection resistance^[34,35]^.

## The imperative of individualized treatment and a holistic approach in long-term management

In managing pancytopenia in HIV-positive individuals with coexisting CHF, the inclusion of an individualized treatment plan is not merely advisable – it is indispensable. This dual-disease burden involves complex pathophysiological interactions, often leading to variable clinical presentations and therapeutic responses. A generalized or rigid management protocol fails to account for the nuanced interplay between immune suppression, cardiovascular dysfunction, and hematologic abnormalities, all of which vary significantly from patient to patient. Hence, individualized care becomes the cornerstone of long-term management, allowing clinicians to tailor therapeutic interventions based on disease severity, comorbid conditions, response to treatment, and patient-specific socioeconomic and psychosocial contexts[17]. Individualized treatment begins with a thorough clinical assessment that appreciates the dynamic presentation of pancytopenia in the backdrop of HIV and CHF. Symptoms such as fatigue, breathlessness, or recurrent infections may be multifactorial and require careful interpretation. For instance, dyspnea in such patients could stem from anemia-induced hypoxia, fluid overload due to poor cardiac output, or pulmonary infections linked to neutropenia. Only a personalized clinical assessment can tease out these causes and lead to appropriately targeted interventions. Moreover, the selection and adjustment of medications – whether antiretroviral therapy, heart failure drugs, or hematopoietic agents – must consider patient-specific risks, such as potential marrow toxicity, renal clearance, or drug-drug interactions. For example, switching from zidovudine to a less myelotoxic ART may benefit a patient with declining blood counts, while cautious titration of diuretics in CHF patients with borderline renal function may prevent worsening cytopenias^[30,31]^.

Equally vital is the integration of a holistic approach that addresses more than the physiological aspects of disease. Holistic care recognizes that long-term health outcomes are shaped not only by clinical interventions but also by nutritional status, mental well-being, social support, and health literacy. Nutritional deficiencies – particularly iron, folate, and vitamin B12 – are common in this population and can exacerbate cytopenias. Addressing these through dietary counseling or supplementation supports hematologic recovery and overall well-being. Mental health is another crucial domain, as depression, stigma, and chronic disease burden can significantly affect adherence to both ART and CHF regimens. Providing psychological support, peer counseling, and psychiatric care, when necessary, can enhance treatment engagement and long-term resilience^[32,33]^. Furthermore, the role of social determinants of health cannot be overstated. Financial instability, poor access to healthcare, lack of transportation, and low health literacy may impede follow-up visits, regular monitoring, and medication adherence. An individualized plan that incorporates the services of social workers, community health workers, and patient navigators can mitigate these challenges, fostering continuity of care. A holistic model also facilitates the inclusion of family and caregivers in the care process, ensuring that patients receive the support they need outside the clinical setting^[34,35]^.

## Conclusion

Pancytopenia in HIV-positive patients with CHF presents a complex clinical challenge due to the interplay of viral infection, heart dysfunction, medication side effects, bone marrow suppression, and immune dysregulation. The mechanisms underlying pancytopenia in this population include HIV-induced bone marrow suppression, CHF-related hypoxia and inflammation, nutritional deficiencies, opportunistic infections, and drug toxicity. Effective management requires a multifaceted approach, addressing the primary causes while ensuring optimal hematological, cardiac, and immune function. Diagnosis of pancytopenia in HIV-CHF patients relies on comprehensive hematological and biochemical evaluations, including CBC, bone marrow biopsy, viral and bacterial infection screening, and cardiac function tests. Early identification of causative factors enables timely interventions, reducing complications such as severe anemia, recurrent infections, and bleeding disorders. Given the potential overlap between medication-induced cytopenias and disease-related marrow suppression, frequent monitoring is critical for guiding treatment adjustments.

## Data Availability

Not applicable as this a narrative review.

## Research registration unique identifying number (UIN)

Not applicable as this a narrative review.
